# Comparison of fine-needle cytologic diagnosis between the left and right liver lobes of dogs and cats with diffuse liver disease

**DOI:** 10.14202/vetworld.2021.2670-2677

**Published:** 2021-10-21

**Authors:** Nardtiwa Chaivoravitsakul, Katriya Chankow, Kongthit Horoongruang, Luksamee Limpongsai, Artima Tantarawanich, Latticha Pluemhathaikij, Kasem Rattanapinyopituk, Kris Angkanaporn

**Affiliations:** 1 Small Animal Teaching Hospital, Faculty of Veterinary Science, Chulalongkorn University, Bangkok, Thailand; 2 Department of Veterinary Pathology, Faculty of Veterinary Science, Chulalongkorn University, Bangkok, Thailand; 3 Department of Veterinary Physiology, Faculty of Veterinary Science, Chulalongkorn University, Bangkok, Thailand.

**Keywords:** aspiration, cytology, diffuse, hepatic, liver, ultrasound

## Abstract

**Background and Aim:**

Ultrasound-guided fine-needle sample collection for cytology with manual restraint is frequently used for the primary assessment of diffuse liver disease in veterinary patients in Thailand. For better diagnosis, repeated collection of samples ensures the collection of adequate, representative samples, which increase diagnostic accuracy. However, in those that are unable to receive general anesthesia, it is difficult to collect the samples from several liver locations in manually restrained dogs and cats. The study aimed to compare the cytologic diagnosis of the ultrasound-guided fine-needle non-aspiration technique between the left and right liver lobes in dogs and cats with neoplastic and non-neoplastic diffuse liver disease.

**Materials and Methods:**

This prospective study included 25 client-owned dogs and cats with diffuse liver diseases. Two liver samples were randomly collected from the left and right liver lobes under ultrasound guidance for cytologic examination. All slides were subsequently examined blindly by experienced pathologists for cytologic analysis with cytologic agreement scores (CASs).

**Results:**

Among all 50 samples obtained from ultrasound-guided fine-needle sample collection of the left and right liver, 78% were diagnostic and 22% were non-diagnostic. In the diagnostic group, 73.3% of fine-needle samples had concordant results between the left and right liver, which exhibited 100% cytologic agreement in lymphoma and 63.6% in non-neoplastic groups. Samples collected from the left liver had slightly higher CAS and higher cytologic quality than had those from the right liver lobe (p=0.053).

**Conclusion:**

The location and number of sample collections did not have a significant difference in the cytologic diagnosis of diffuse liver disease, especially in patients with lymphoma. For manually restrained patients, one time ultrasound-guided non-aspiration cytology procedure from the left liver lobe not only decreased restraint duration and minimized tissue trauma but also allowed for an adequate cytologic diagnosis in diffuse liver disease compared to multiple collections.

## Introduction

The liver is a vital organ in both animals and humans. It plays an important role in the biological processes of the body, including homeostasis, digestion, and detoxification [1-3]. Liver disease is a common problem encountered in veterinary medicine. It manifests with variable clinical signs such as icterus, ascites, coagulopathy, and hepatic encephalopathy and can also cause non-specific signs such as anorexia, vomiting, and diarrhea [1]. Liver disease is diagnosed by analyzing a combination of the patient’s characteristics: Serum biochemistry profiles, coagulation profiles, urinalysis, imaging, and hepatic tissue evaluation [1-5]. Ultrasonography is a highly efficient diagnostic tool for liver disease [6-8]; it is commonly used in veterinary medicine to assess liver disease in combination with serum biochemistry. The ultrasonographic features of the liver in patients with liver disease may be variable. There could be alterations in the echogenicity, size, and contour of the liver, or it may even appear to be normal [9]. Based on ultrasonographic findings, liver diseases can be categorized according to its distribution as diffuse parenchymal disease, focal or multifocal parenchymal disease, vascular disease, and biliary tract disease [7,8]. Diffuse parenchymal abnormality is usually an infiltrative disease [3], including vacuolar hepatopathy, hepatic lipidosis in cats [6], hepatitis, amyloidosis, cirrhosis or fibrosis, and some neoplastic diseases (e.g., lymphoma [3] and metastatic mast cell tumor [10]). Regardless of the category of liver disease (i.e., diffuse, focal, or multifocal), liver lesions have no specific ultrasonographic features [11,12], and thus, a definitive diagnosis cannot be achieved using ultrasound alone. Liver biopsy remains the gold standard for liver disease diagnosis, but this procedure requires general anesthesia, is more expensive, and is associated with potentially life-threatening complications, especially in critically ill and geriatric patients. Moreover, owners of these patients often refuse these procedures to avoid their anesthetic risks, which then make the diagnosis inconclusive and specific treatment impossible. Ultrasound-guided fine-needle sample collection for cytology is used as a primary assessment of liver disease in human and veterinary patients because of its simplicity, speed, and low risk for complications [13-16] and remains the most commonly used staging technique for metastatic lymphoma [17]. Aspiration of the liver is recommended for the detection of lymphoma regardless of its ultrasonographic appearance [18]. The fine-needle non-aspiration technique was found to be superior to the aspiration technique [19,20]. Ultrasound-guided fine-needle sampling of the liver requires at least two to three aspirates randomly collected from the liver to increase diagnostic accuracy, and it is routinely performed from the left lateral or left medial lobe liver [6].

However, patients that are unable to receive sedation or general anesthesia because of restriction on either anesthetic risks or financial problems. This frequently occurs in routine clinical practice cases in Thailand; hence, it is difficult to collect samples from several liver locations. As a consequence of this limitation, concordant results between numbers and locations of liver sampling may be of significant clinical importance.

The study aimed to compare single and multiple ultrasound-guided fine-needle sample collection in terms of cytologic diagnostic results between the left and right liver lobes of dogs and cats with neoplastic and non-neoplastic diffuse liver disease. We hypothesized that the cytologic diagnosis through ultrasound-guided fine-needle sample collection had no significant difference between collection from the left liver (which is routinely performed) and the right liver in the patients with diffuse liver disease in both neoplastic and non-neoplastic groups. The cytologic quality of ultrasound-guided fine-needle sample collection was also compared between these two liver locations.

## Materials and Methods

### Ethical approval and Informed consent

The study was exempted for consideration in the institutional animal care and use committee because the diagnostic procedures were a part of the clinical diagnostic workup in routine clinical practice. Written consent was obtained from all owners to use the animals for clinical examination.

### Study animals, period, and location

A total of 25 client-owned patients were prospectively recruited from university’s small animal teaching hospital from June 2020 to December 2020. The inclusion criteria were dogs and cats of any age or body weight with either suspected liver disease based on clinical findings and laboratory results (at least one liver enzyme elevation) or undergoing staging for lymphoma spread to liver. In addition, all patients should have ultrasonographic results consistent with diffuse liver disease that requires ultrasound-guided fine-needle sample collection of liver cytology as part of their clinical diagnostic workup. The exclusion criteria were patients with focal or multifocal liver lesions, with small liver size based on radiographic examination, or unable to undergo ultrasound-guided fine-needle sample collection of fine-needle cytology. Patients with clinical signs relevant to coagulopathy (e.g., nasal bleeding and hematochezia) that can elicit high risk of complication were also excluded from this study. Serum liver enzyme activity, specifically alanine aminotransferase (ALT) and alkaline phosphatase (ALP), was measured before abdominal ultrasonography in all patients. Increased liver enzyme were classified as either mild (2-3 times elevation in activity), moderate (5-10 times elevation in activity), or marked (>10 times elevation in activity).

### Ultrasonography and sample collection

Ultrasound examination was performed for the detection of diffuse liver disease using a Logiq P6 (GE Healthcare, Milwaukee, USA) and 6-10 MHz curvilinear or 11-13 MHz linear electronic transducer. All patients were examined in dorsal recumbency and restrained manually with tranquilization when necessary. Percutaneous fine-needle sample collection of fine-needle cytology was performed in all patients during abdominal ultrasonography. Diffuse liver disease was defined by the characteristics of the liver parenchyma in terms of uniformity (diffuse homogeneous or diffuse heterogeneous) and echogenicity relative to spleen (hypoechoic or hyperechoic). Other liver ultrasound findings were also recorded, including liver size (normal, increased, or decreased) and margin (normal, rounded, or irregular).

Fine-needle sample collection of fine-needle cytology of liver was performed with a 1.5 in. 24 G or 1 in. 26 G needle attached to a 3 mL syringe under ultrasound guidance. In each patient, two liver samples (one each from the left and right lobes) were randomly collected for cytologic examination by the same radiologist using the same collecting method and equipment. Sampling was performed without aspiration [21], with four to five short and rapid strokes within each lesion. The obtained slides were air-dried and stained through Giemsa stain. Then, 50 slides from 25 patients were randomly shuffled and relabeled from 1 to 50 by an external technician. These were examined by three experienced pathologists in a blinded fashion; cases were diagnosed without previous knowledge of the interpretation, species, and location.

### Cytologic analysis

**Figure-1:**
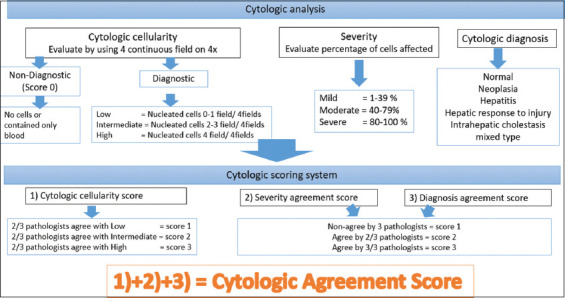
The cytologic analysis and scoring system flowchart.

The cytologic analysis was independently assessed following the steps described in Figure-1. We evaluated cytologic cellularity, severity agreement, and diagnostic agreement and then finally determined the cytologic agreement score (CAS) of the left and right liver samples from each patient. The cytologic result was determined as non-diagnostic if there were no cells or contained only blood and was scored as 0 in both severity agreement and diagnosis agreement. The cytologic severity and diagnostic agreement of each liver sample was finalized based on majority agreement between the three pathologists. CASs of 1-4, 5-7, and 8-9 were considered low, intermediate, and high agreement, respectively (Table-1). The cytologic diagnosis was divided into six groups: (1) Normal; (2) neoplasia; (3) hepatitis; (4) hepatic response to injury, including degeneration, regeneration, fibrosis, and bile duct hyperplasia; (5) intrahepatic cholestasis; and (6) mixed type, including hepatitis with intrahepatic cholestasis or hepatic response to injury with intrahepatic cholestasis [22].

**Table 1:** Criteria for determining cytologic agreement score (cellularity+severity agreement+diagnosis agreement) of patients.

Cytologic agreement score	Level of agreement
1-4	Low agreement
5-7	Intermediate agreement
8-9	High agreement

### Statistical analysis

Descriptive statistics were used to analyze all parameters. The Chi-square test was used to analyze the CAS of both sides of liver. The Mann–Whitney rank-sum test was used to compare the individual CAS and CAS sum score of each patient, as well as the left and right lobe samples. TheSigmaStat 3.5 statistical program (Systat Software, San Jose, CA, USA) was used in the analysis with statistical significance set at p<0.05.

## Results

The 25 patients included 20 dogs (11 males and nine females) and five cats (four males and one female). The dog breeds included Chihuahua (n=4), toy Poodle (n=3), Pomeranian (n=2), mixed breed (n=6), Beagle (n=1), Scottish terrier (n=1), Terrier (n=1), Golden retriever (n=1), and Shih Tzu (n=1). The cat breeds included domestic shorthair (n=3), Persian (n=1), and Scottish fold (n=1). In all patients, the mean±SD of age was 8.81±3.02 years (range: 1-14 years)

**Figure-2:**
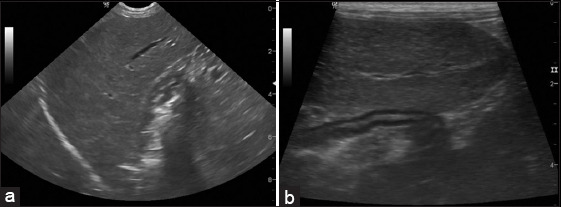
Longitudinal ultrasound images of liver. Microconvex C6-10 MHz (a) and Linear L11-13 MHz (b) images showed diffuse hypoechoic parenchyma with round margin of the liver.

The serum ALT and ALP results are shown in Table-2. The mean serum ALT was 428.56 IU/L (range: 45-3671 IU/L), while the mean serum ALP was 2016.68 IU/L (range: 56-13,706 IU/L). The most common changes were elevation of both liver enzymes in 15 patients, while normal levels of both liver enzymes were found in two dogs. Normal ALT was found in five out of 25 patients, whereas mild, moderate, and marked ALT elevations were found in 15, 3, and 2 patients, respectively. Meanwhile, normal ALP was found in one patient, whereas mild, moderate, and marked ALP elevations were found in 10, 4, and 10 patients, respectively [23]. Out of the 25 patients, ultrasonography revealed a homogeneous echotexture in 64% (16 patients; dog n=13, cat n=3) and a heterogeneous echotexture in 36% (nine patients; dog n=7, cat n=2). The liver was hypoechoic in 36% (nine patients; dog n=7, cat n=2) and hyperechoic in 64% (16 patients; dog n=13, cat n=3). In terms of size, the liver was enlarged in 76% (19 patients; dog n=16, cat n=3), normal in 20% (five patients; dog n=3, cat n=2), and small in 4% (one patient; dog n=1). The liver margin was normal in 28% (seven patients; dog n=4, cat n=3), rounded in 60% (15 patients; dog n=13, cat n=2), and irregular in 12% (three patients; dog n=3). The sonographic images revealing diffuse parenchymal disease with round liver margins are shown in Figure-2, while the relationship between sonographic findings and cytologic diagnosis is shown in Table-3.

**Table 2:** ALT and ALP levels in all patients.

Parameter	Mean (range)	Cutoff value	Patient with normal value, n (%)	Patient with abnormal value, n (%)					

				Mild		Moderate		Marked	
		
				Dog	Cat	Dog	Cat	Dog	Cat
ALT (IU/L)	428.56 (45-3671)	Dog>91	Dog 5/20 (25)	10/20 (50)	—	3/20 (15)	—	2/20 (10)	—
		Cat>75	Cat 0/5 (0)	—	5/5 (100)	—	—	—	—
ALP (IU/L)	2016.68 (56-13706)	Dog>60	Dog 0/20 (0)	6/20 (30)	—	4/20 (20)	—	10/20 (50)	—
		Cat>61	Cat 1/5 (20)	—	4/5 (80)	—	—	—	—
ALT and ALP (IU/L)			Dog 2/20 (10)	—	—	10/20 (50)	—	—	—
			Cat 0/5 (0)	—	—	—	5/5 (100)	—	—

ALT=Alanine aminotransferase, ALP=Alkaline phosphatase

**Table 3:** The relationship between sonographic findings and cytologic diagnosis.

Sonographic finding of diffuse liver disease	Normal (0)	Response to injury (12)	Hepatitis (2)	Neoplasia (4)	Intrahepatic cholestasis (0)	Mixed (2)
Size						
Enlarged		9	2	4		2
Normal		2				
Small		1				
Margin						
Smooth and sharp		3				
Round		8	2	4		1
Irregular		1				1
Parenchymal echogenicity						
Hypoechoic		3	2	1		
Hyperechoic		9		3		2
Parenchymal echotexture						
Homogeneous		8		4		
Heterogeneous		4	2	4		2

Case nos. 9, 11, 15, and 16 were not included in this table because of inconsistency in the cytologic diagnosis. Case no. 23 was included because of the non-diagnostic result from both liver locations.

All patients in the neoplastic group had lymphoma, characterized by an enlarged liver with round margins and a homogeneous echotexture. Hyperechogenicity was seen in three cases, while hypoechogenicity was seen in one. In the hepatitis group, majority of patients had hepatomegaly with heterogeneous hypoechoic parenchyma and round margins. In the hepatic response to injury group, the livers appeared enlarged and had hyperechoic parenchyma with round margins and a homogeneous echotexture. In the mixed group, the livers were enlarged with hyperechogenicity and a heterogeneous echotexture, with either round or irregular margins.

In the analysis of cytologic quality, among a total of 50 samples (25 left liver samples and 25 right liver samples), 78% were diagnosed, while 22% were non-diagnostic (inadequate cell number; left liver n=3, right liver n=4). There were non-diagnostic cases because of blood contamination in one left liver sample and three right liver samples. There was only one dog with non-diagnostic samples from both the left and right liver. Regarding the cytologic diagnosis of samples obtained from 25 patients (Table-4), there were 15 patients with adequate cells for cytologic diagnosis from both liver locations. Among these 15 patients, 11 (73.3%) had concordant cytologic results between the left and right liver. In the cytologic diagnosis divided by groups, all four patients in the neoplastic group (100%) had concordant results between the left and right liver.

**Table 4:** Cytologic diagnosis of the left liver and right liver obtained by ultrasound-guided fine-needle sample collection.

Cytologic diagnosis	Obtained cytologic diagnosis from two liver locations (n=15)			Obtained cytologic diagnosis from one liver location (n=9)	
	
	Cytologic diagnostic agreement (n)	Cytologic diagnostic disagreement (n)	Percentage of diagnostic consistency n (%)	Cytologic diagnosis from left liver (n)	Cytologic diagnosis from right liver (n)
Normal (n=0)	0	0		0	0
Neoplastic group (n=4)	4	0	4 (100)	0	0
Lymphoma	4	0		0	0
Non-neoplastic group (n=20)	7	4	7 (63.6)	6	3
Hepatitis	1			0	1
Hepatic response to injury	5			6	1
Intrahepatic cholestasis	0			0	0
Mixed	1			0	1
Total (n=24)*	11	4	11 (73.3)	6	3

* n=24, excluded one dog with no diagnosis

Of all 20 patients in the non-neoplastic group, 11 obtained a cytologic diagnosis from both liver locations. Among these, 7 (63.6%) had concordant results between liver locations (response to liver injury: 5/7 [71.4%]; hepatitis: 1/7 [14.2%], and mixed group: 1/7 [14.2%]). By contrast, four had discordant results as follows: Mixed type in the left liver and intrahepatic cholestasis in the right liver (n=1), mixed type in the left liver and hepatitis in the right liver (n=1), intrahepatic cholestasis in the left liver and mixed type in the right liver (n=1), and response to liver injury in the left liver and mixed type in the right liver (n=1).

**Figure-3:**
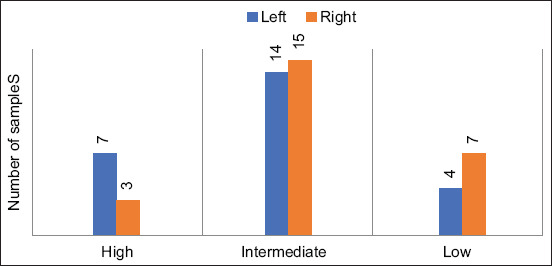
Cytologic agreement score of individual left and right liver samples.

**Figure-4:**
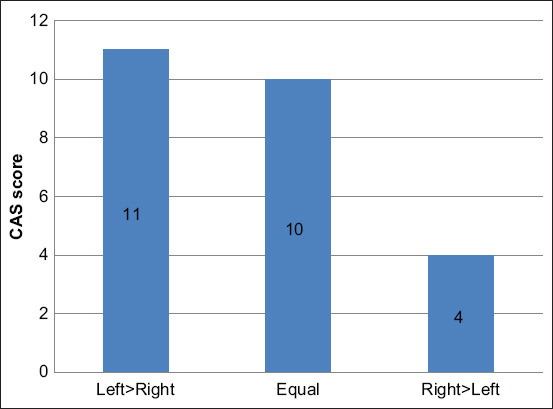
Comparison of cytologic agreement score of each animal.

The sum of CASs of all samples from the left and right liver is depicted in Figure-3 and Table-5. Of the samples obtained from the left liver, 28% (7/25) had high CAS, 56% (14/25) had intermediate CAS, and 16% (4/25) had low CAS. Of the samples obtained from the right liver, 12% (3/25) had high CAS, 60% (15/25) had intermediate CAS, and 28% (7/25) had low CAS. These results showed no significant difference (p>0.05). The CASs of the left and right liver of each animal were compared (Figure-4). There were 44% (11/25) that had higher CAS in the left liver, 10% (10/25) had equal CAS in both sides, and only 16% (4/25) had higher CAS in the right liver.

**Table 5:** Cellularity score, severity score, diagnosis agreement score, and cytologic agreement score (CAS) sum score of the left and right lobes of liver.

Cytologic analysis	Left liver (n=25)				Right liver (n=25)			
	
	Cellularity score, n (%)	Severity score, n (%)	Diagnosis agreement score, n (%)	Sum of CAS score, n (%)	Cellularity score, n (%)	Severity score, n (%)	Diagnosis agreement score, n (%)	Sum of CAS score, n (%)
High/severe	7 (28)	15 (60)	15 (60)	7 (28)	5 (20)	10 (40)	10 (40)	3 (12)
Intermediate/moderate	9 (36)	6 (24)	6 (24)	14 (56)	8 (32)	7 (28)	8 (32)	15 (60)
Low/mild	9 (36)	0	0	4 (16)	12 (48)	1 (4)	0	7 (28)
No diagnosis	0	4 (16)	4 (16)	-	0	7 (28)	7 (28)	-
Total score (average, range)	1.92 (0-3)	2.28 (0-3)	2.28 (0-3)	6.48 (0-9)	1.72 (0-3)	1.8 (0-3)	1.84 (0-3)	5.34 (0-9)

When the sum of the CASs was compared between the right and left lobes (Table-5), the left lobe had a slightly higher CAS sum score than had the right lobe (6.48±0.51 vs. 5.36±0.57, respectively, p=0.053). All other parameters, including cellularity score, severity score, and diagnosis agreement score were not significantly different (p>0.05) between the two sides.

## Discussion

In this study, ultrasound-guided fine-needle liver sampling for cytology in diffuse liver disease had no significant difference in cytologic diagnosis between samples obtained from the left and right liver. There was 73.3% cytologic diagnosis agreement between both locations of fine-needle sample collection. In the patients with liver metastasis from lymphomas, there was 100% agreement between the left and right liver samples, with an intermediate to high CAS. It is well known that hemolymphatic lymphoma metastasis causes diffuse infiltration of the neoplastic cells in the liver parenchyma [19], and lymphoma can be reliably diagnosed cytologically [24,25]. In addition, hepatomegaly caused by neoplastic cell infiltration may also increase accessibility when performing ultrasound-guided fine-needle liver sampling for cytology. This suggests that in lymphoma cases, sample collection from only one location with adequate diagnostic cells may be sufficient for the diagnosis of metastatic lymphoma to the liver.

Four patients had disagreements in cytologic diagnosis between the left and right liver samples. These patients were in the non-neoplastic diffuse liver disease group, which had various disease processes occurring within the liver and had no specific differences in ultrasonography [9]. Hepatitis could be detected in combination with other lesions such as degeneration, fibrosis, cholestasis, and nodular hyperplasia [26]. Moreover, fibrosis can be a consequence of many inflammatory conditions; it may occur as porto-portal, porto-central, centro-central, or lobular dissecting fibrosis [27]. The nature of the lesion and distribution of representative cells were thought to be the main reasons for variations in concordance. A previous study suggested that in case of chronic lymphocytic hepatitis in dogs and cholangiohepatitis in cats, the inflammatory cells are located in the periportal areas and in dense connective tissue, rather than being distributed throughout the liver parenchyma. Therefore, the areas of inflammation may escape detection from cytologic examination [26,28]. In this study, discordances in cytologic diagnosis occurred when the diagnoses were mixed type in one side and either response to injury, intrahepatic cholestasis, or hepatitis on the other side. In these cases, one side was diagnosed as mixed type due to the additional diagnosis of intrahepatic cholestasis along with findings similar to those of the other side. For example, a left liver sample was diagnosed as mixed type (with findings of hepatitis and intrahepatic cholestasis), while its corresponding right liver sample was diagnosed with hepatitis. Previous publications in veterinary medicine describe cholestasis as a unique cytologic feature of fibrosis [29]. Then, it is likely that intrahepatic cholestasis does not occur evenly throughout the liver and might have been missed during sample collection. However, in these cases, intrahepatic cholestasis was diagnosed by only one out of three pathologists (excluded for final diagnosis), which may also suggest subjective factors involved in the diagnosis. However, the diagnostic samples were all categorized as having high or intermediate CAS.

All non-diagnostic samples had low CAS. The fine-needle sample collection using a non-aspiration technique showed ineffective sample collection of the liver, with 22% of non-diagnostic samples caused by blood contamination, which correlated with the previous study [19]. In our study, 36% of cases had only one diagnostic sample from a single location (either left or right liver), and non-diagnostic samples were more likely to be collected from the right liver (66.6%). Out of all 50 liver samples, those collected from the left liver had more cytologic cellularity and greater cytologic diagnosis agreement scores than those from the right liver. It is logical that the left liver samples had higher quality of cells than the right liver samples because of easier accessibility, considering its anatomical location. Moreover, there were also many factors affecting the number of cells collected, including the size of the needle, patient compliance, and number of strokes during sample collection, and larger needle diameter produced greater tissue yields [29].

The limitations of this study include its low sample size and lack of variation in the neoplastic group. This study was performed during routine clinical practice, and the radiologists were not aware of inadequate samples before ending the treatment. General anesthesia could not be performed in this study because of either concern about anesthetic risks or financial problems, and thus, radiologists were unable to collect samples from more liver locations for comparisons. As mentioned in several previous publications, the gold standard for liver disease diagnosis is liver biopsy [9,30]; thus, the confirmation of the diagnosis remains unknown.

## Conclusion

In diffuse liver diseases, the location of sample collection did not have a significant difference in cytologic diagnosis in patients with lymphoma and non-neoplastic diffuse liver disease. Samples from the left liver had higher CAS sum scores than had those from the right liver, possibly because of higher cytologic cellularity. For patients needing manual restraint, the one time ultrasound-guided non-aspiration cytology procedure from the left liver lobe can provide adequate cytologic diagnosis compared to collections from multiple locations, especially in diffuse liver disease patients. This has the added benefit of decreasing the duration of restraint and minimizing liver tissue trauma. For further studies, we suggest fine-needle liver sampling from several different locations, in either the same or different liver lobes to correlate this with the cytologic diagnosis. We also recommend increasing variability of cases with neoplastic diffuse liver disease, as well as include liver biopsy results or necropsy results, if available, to confirm the diagnosis.

## Authors’ Contributions

NC: Designed the overall study, performed the ultrasonography, collected the cytological samples, and drafted the manuscript. KH: Performed the ultrasonography, collected the cytologic samples, and assisted in the manuscript writing. LL and AT: Performed the ultrasonography and collected the cytologic samples. KC: Designed the study, performed the cytologic diagnosis, and assisted in the manuscript writing. LP: Designed the pathological study and performed the cytologic diagnosis. KR: Performed the cytologic analysis and assisted in the manuscript writing. KA: Designed the study, conducted the statistical data analysis, and edited the manuscript. All authors read and approved the final manuscript.
